# Laser Phototherapy (660 nm) Can Be Beneficial for Reducing Gingival Inflammation in Prosthodontics

**DOI:** 10.1155/2015/132656

**Published:** 2015-09-30

**Authors:** Sávio José Cardoso Bezerra, Glauco Fioranelli Vieira, Carlos de Paula Eduardo, Patrícia Moreira de Freitas, Ana Cecilia Corrêa Aranha

**Affiliations:** ^1^Department of Restorative Dentistry, School of Dentistry, University of São Paulo, Avenida Professor Lineu Prestes 2227, 05508-000 São Paulo, SP, Brazil; ^2^Special Laboratory of Lasers in Dentistry, Department of Restorative Dentistry, School of Dentistry, University of São Paulo, Avenida Professor Lineu Prestes 2227, 05508-000 São Paulo, SP, Brazil

## Abstract

Among the new technologies developed, low power lasers have enabled new approaches to provide conservative treatment. Low power lasers act at cellular level, resulting in reduced pain, modulating inflammation, and improved tissue healing. Clinical application of the low power laser requires specific knowledge concerning laser interaction with biological tissue so that the correct irradiation protocol can be established. The present case report describes the clinical steps involved in an indirect composite resin restoration performed in a 31-year-old patient, in whom low power laser was used for soft tissue biomodulation. Laser therapy was applied with a semiconductor laser 660 nm, spot size of 0.028 cm^2^, energy density of 35.7 J/cm^2^, mean power of 100 mW, and energy per point as 1 J, in contact mode, on a total of 2 points (mesial and distal), totaling 2 J of energy. The therapy with low power laser can contribute positively to the success of an indirect restorative treatment.

## 1. Introduction

Periodontal disease is defined as an “inflammatory disease of supporting tissues of the teeth caused by specific microorganisms, which leads to progressive destruction of the periodontal membrane and alveolar bone, with formation of periodontal pockets and gingival recession.” [1]. The treatment of gingivitis (inflammation confined to the gingival tissues) and periodontitis includes various stages. These range from the simplest, classical treatment methods (cleaning and scaling), through to more invasive interventions (surgeries), and, recently, to more conservative and adjunct treatments such as the use of low power lasers [2].

Low power laser irradiation may have both a local and general anti-inflammatory effect [2]. The following are some of the characteristics of this therapy: it induces an increase in nonspecific protection factors such as the complement system [3], interferon, and others [4]; reduces interstitial and intracellular swelling by improving blood circulation in the damaged tissue, often resulting in less pain at the site of injury [5]; decreases in the levels of histamine and serotonin, which are mediators in the inflammation process and also have vasodilation effects [6, 7]; decreases in the porousness of blood vessels, preventing alteration-exudative processes, thereby preventing swelling [8].

The anti-inflammatory effects of low power laser irradiation are observed by a decrease in the number of clinical signs of inflammation, lower acute-phase protein levels, and decrease in circulating immune complexes [8]. In dentistry, low power laser therapy has mainly been used for pain relief in oral mucous membranes, dentin hypersensitivity, and other conditions requiring an analgesic and anti-inflammatory response [8–10]. Its effects on periodontal therapy are known and have been recognized in* in vitro* [11–13] and clinical [14, 15] studies; however, they still need further exploration.

In addition to the benefits achieved by laser therapy in periodontics, its effects are directly related to restorative dentistry and have contributed to the clinical success of restorative procedures [16]. Low power lasers have been widely studied and reports have shown that they offer many benefits in the different steps of direct and indirect restorative treatments, such as in tooth preparation, fabrication of provisional crowns, impressions, and cementation [17].

One of the main goals of the restorative procedure is to provide a healthy gingiva surrounding the tooth preparation in order to avoid postoperative gingival tissue reactions, such as the exposure of the cervical preparation margin that would completely compromise the esthetics [16].

Therefore, the use of laser phototherapy associated with a strict oral hygiene implemented by the patient will lead to healthy periodontal tissue and contribute not only to more adequate luting of the restoration, by avoiding an inflammatory process and bleeding during impression-taking, but also to a more advanced esthetic end result and longevity of the indirect restoration [17].

## 2. Case Presentation

**Figure 1:**
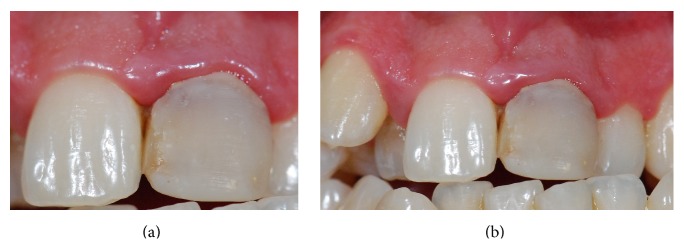
Intraoral exam revealed an inadequate veneer made with composite resin on the right central incisor and recurrent gingivitis on superior anterior teeth.

The patient A. R. M., a 31-year-old man, was referred to the Post-Graduation Clinic of the School of Dentistry of University of São Paulo for treatment, reporting dissatisfaction with the esthetic appearance of a restoration on the right central incisor. After anamnesis, the oral exam revealed an inadequate composite resin veneer placed on the right central incisor and recurrent gingivitis of the maxillary anterior teeth (Figures 1(a) and 1(b)). In addition, a radiographic exam was performed, showing that the right central incisor received a satisfactory endodontic treatment. Treatment of the gingivitis and replacement of the inadequate veneer were then indicated.

**Figure 2:**
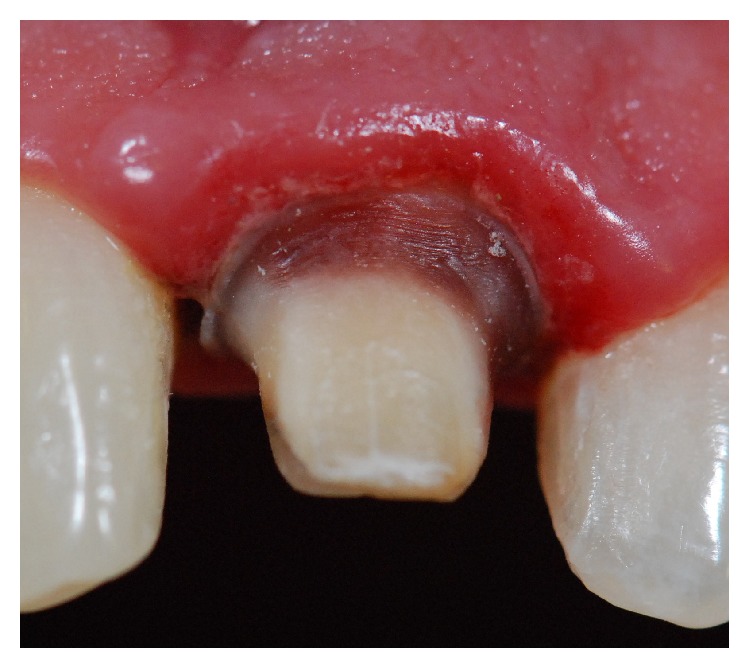
Remaining dental structure was evaluated and it was decided to restore it with a composite resin crown.

**Figure 3:**
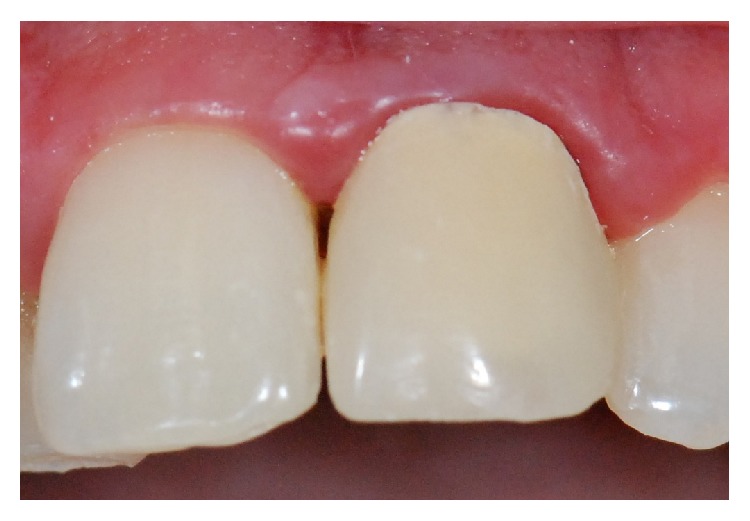
Provisional acrylic-resin crown placed.

The first clinical step was to instruct the patient with regard to healthy oral hygiene habits. These instructions were repeated in all clinical sessions throughout the restorative treatment. After this, the veneer was removed and the remaining dental structure was evaluated, showing that, apart from its severe discoloration, this structure was insufficient to support a veneer. Thus the decision was to restore it with a composite resin crown (Figure 2). The tooth was prepared and a provisional acrylic-resin crown (Jet, color 62, Classico, São Paulo, SP, Brazil) was placed (Figure 3).

**Figure 4:**
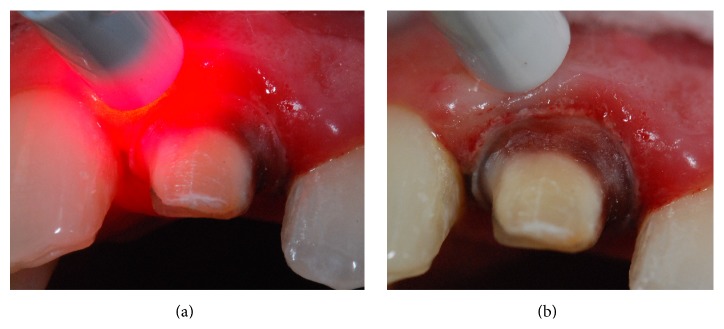
After tooth preparation, laser phototherapy with a low power laser (660 nm) was performed in all clinical sessions (total of four sessions), on the surrounding periodontal tissue (punctually with a semiconductor laser, wavelength of 650 nm, spot size of 0,028 cm^2^, energy density of 35.7 J/cm^2^, mean power of 100 mW, and energy per point as 1 J, contact mode, in a total of 2 points totalizing 2 J of energy).

**Figure 5:**
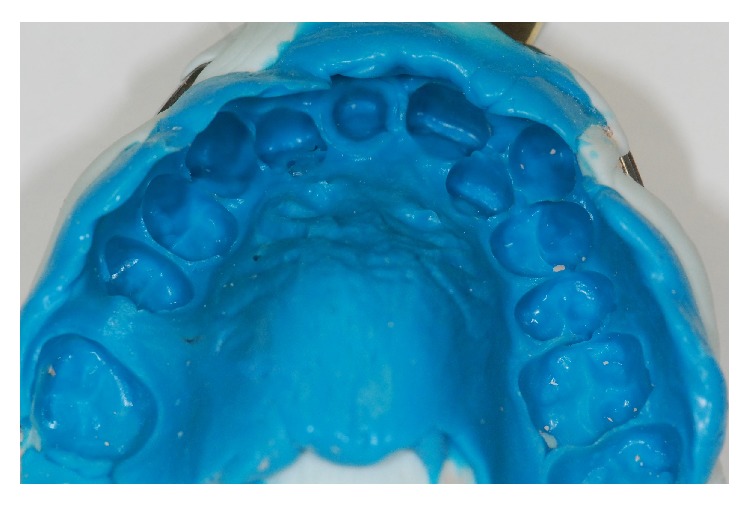
Teeth impression with vinyl polysiloxane impression material.

After tooth preparation, laser phototherapy with a low power laser (660 nm) was performed (Figures 4(a) and 4(b)) on the surrounding periodontal tissue, in all clinical sessions (total of four sessions), until the final luting of the indirect resin crown. Time interval between laser applications was one week. Laser phototherapy was conducted punctually with a semiconductor laser (Photon Lase I, DMC, São Carlos, SP, Brazil) (Figure 5), wavelength of 660 nm, spot size of 0.028 cm^2^, energy density of 35.7 J/cm^2^, mean power of 100 mW, and energy per point as 1 J, in contact mode, on a total of 2 points (mesial and distal), totaling 2 J of energy.

Low power laser phototherapy was carried out with the purpose of promoting soft tissue biomodulation, because both hard and soft tissues needed to be completely healthy at the end of treatment, so that a fully esthetic outcome could be achieved. Likewise, no inflammatory signs should be present in the gingival tissue before final luting procedure.

**Figure 6:**
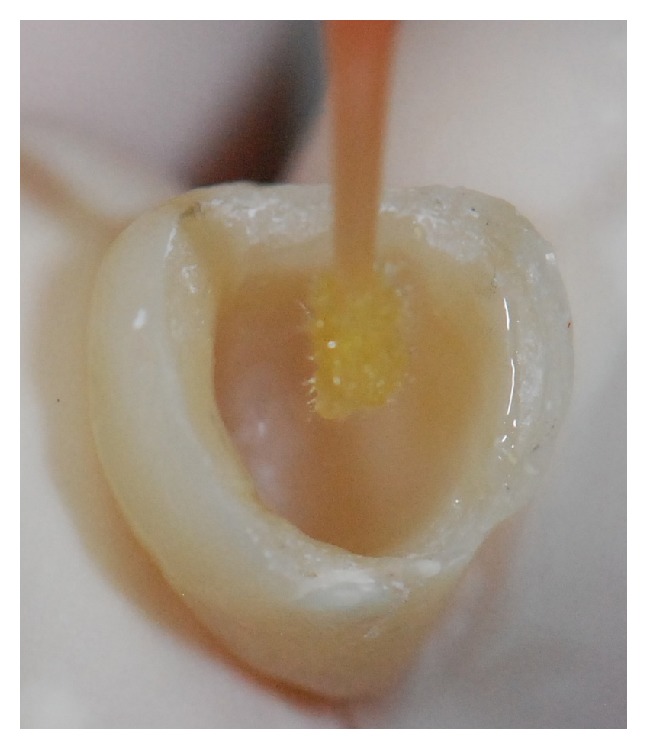
A thin layer of adhesive system was applied using a disposable brush.

**Figure 7:**
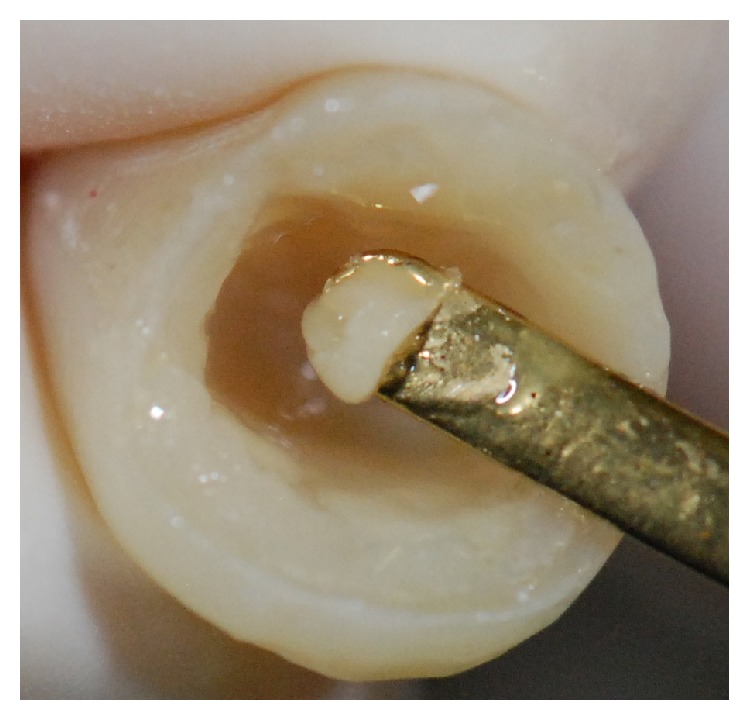
A thin layer of composite resin was applied in the inner surface of the resin restoration, for luting.

**Figure 8:**
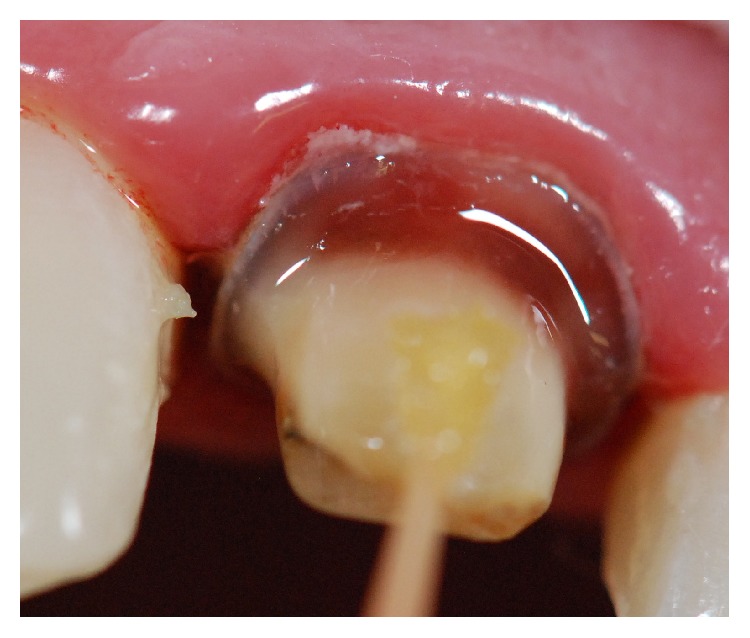
A thin layer of bond was applied with a disposable brush on the entire tooth surface starting with the enamel and applied with slight pressure for 15 seconds, according to the manufacturer's instructions.

**Figure 9:**
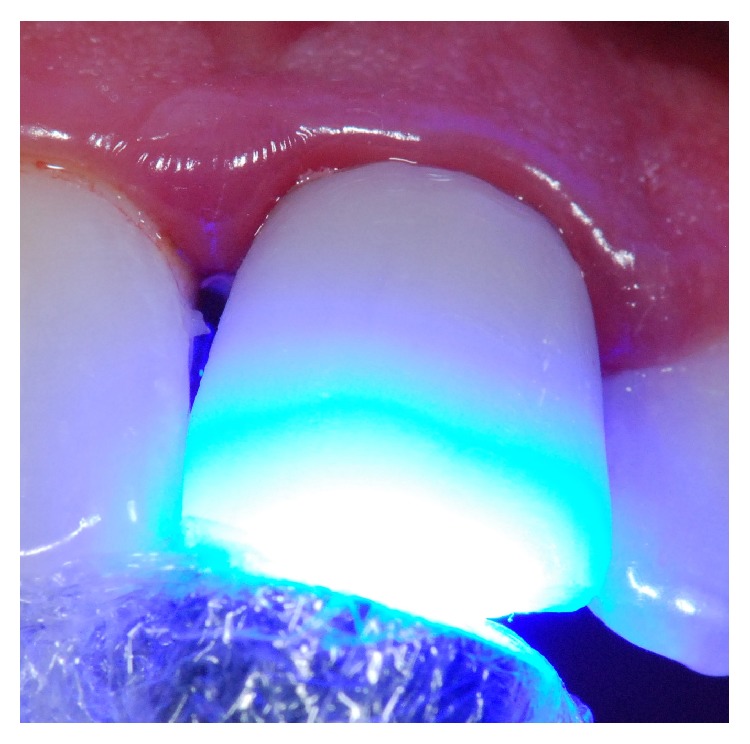
Light activation was carried out for the composite resin for 20 seconds, in lingual and buccal faces.

**Figure 10:**
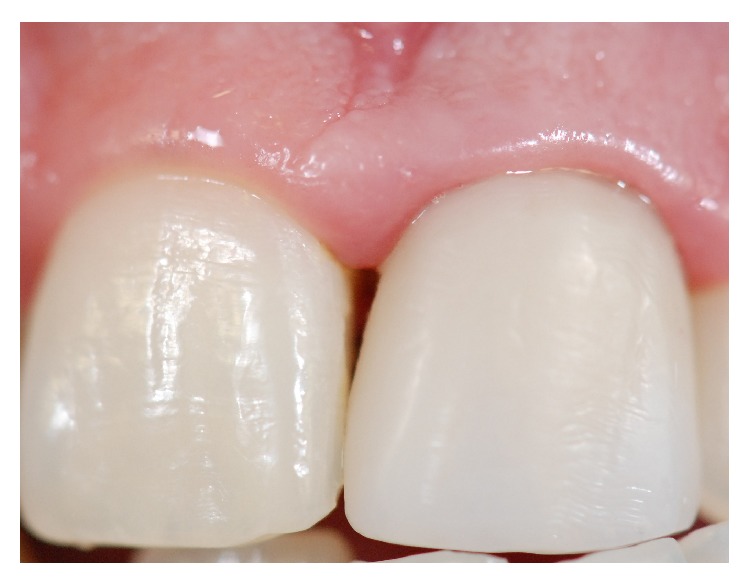
Finally, the occlusion was checked, with articulating paper, and restoration was concluded.

The resin color was selected with a specific color shade guide (Venus Shade Guide, Heraeus Kulzer, Hanau, Germany) and impression of the teeth was taken with vinyl polysiloxane impression material (Perfil, Vigodent Coltene, Rio de Janeiro, RJ, Brazil) (Figure 5). Before final luting, the temporary restoration was removed and the prepared tooth was cleaned to remove any temporary cement. The tooth was rinsed with water and gently air-dried. Subsequently, the indirect resin restoration was proved and the shade and marginal adaptation of the resin restoration were checked. The tooth surfaces and the inner surface of the resin restoration were treated and prepared for adhesive luting. Tooth surface was etched with 35% phosphoric acid (Scotchbond Etchant Phosphoric Acid, 3M ESPE, St. Paul, USA), for 15 seconds, and then abundantly rinsed with water. On the inner surface of the resin restoration (Venus, Heraeus Kulzer, Hanau, Germany), a thin layer of adhesive (Single Bond, 3M ESPE, St. Paul, USA) was applied with a disposable brush (KG Brush, KG Sorensen, São Paulo, SP, Brazil) (Figure 6) and gently air-dried. A thin layer of composite resin (Venus, Heraeus Kulzer, Hanau, Germany) was applied to the inner surface of the resin restoration, for luting (Figure 7). A thin layer of adhesive (Single Bond, 3M ESPE, St. Paul, USA) was applied with a disposable brush (KG Brush, KG Sorensen, São Paulo, SP, Brazil) on the entire tooth surface (Figure 8), starting with the enamel, applying slight pressure for 15 seconds, according to the manufacturer's instructions. The restoration was seated in place and held. Excess composite resin was immediately removed. The composite resin was polymerized by light activation (Optilight Max, Gnatus, Ribeirão Preto, SP, Brazil) for 20 seconds on the lingual and buccal surfaces (Figure 9). Finally, the occlusion was checked with articulating paper (Accufilm, Parkell Inc., New York, USA). The concluded case may be observed in Figure 10.

## 3. Discussion

Laser phototherapy has shown satisfactory results as an adjunct therapy to control gingival tissue healing by biomodulation [17, 18].

After 1971, when Mester et al. [19] first reported the biological effects and benefits of low power lasers, this therapy modality has been considered an alternative and noninvasive method to enhance chronic wound healing, modulate the inflammatory process, and promote pain relief [11, 20–25].

At a cellular level, light is absorbed by specific chromophores, and once absorbed, the light can modulate cell biochemical reactions and stimulate mitochondrial activity [26]. This primary answer will lead to secondary responses, such as increase in ATP synthesis; collagen production; increase in cell proliferation and migration; and biomodulation of inflammatory processes [27, 28].

Clinical studies have reported the effects of laser phototherapy on the modulation of periodontal inflammatory process, verifying that patients who received conventional periodontal treatment associated with laser phototherapy presented better results than nonirradiated patients [14, 29].

Ozawa et al., 1997 [30], showed that laser phototherapy significantly inhibited the increase in plasminogen activity induced in human periodontal ligament cells in response to mechanical tensile force. Plasminogen activity is capable of activating latent collagenase, the enzyme responsible for cleaving collagen fibers.

An* in vivo* study conducted by Pejcic et al. [15] evaluated the effects of low-level laser irradiation treatment and conservative treatment on gingival inflammation. Authors reported a statistically significant improvement in the gingival index and bleeding index after laser irradiation, especially over a longer period of time (3 and 6 months after irradiation). The authors concluded that low power laser irradiation (670 nm) associated with traditional periodontal therapy could lead to better and longer-lasting therapeutic results. In the present clinical case, the patient showed an improvement in gingival inflammation after laser irradiation, as observed in Figure 10.

The frequency of the laser phototherapy sessions is one of the factors that can influence the success of the treatment. Some studies have shown that a single irradiation is not as effective as successive sessions [31, 32]. The transitory effects in cells resultant from laser phototherapy have also been demonstrated in the literature, suggesting that the number of sessions is important to maintain the laser effect on cells [15]. However, as it is sometimes difficult for the patient to return to the dental office to perform 10 consecutive laser sessions, the objective of this study was to show that the dentist can perform the irradiation when the patient returns, having laser irradiation as part of the procedure, considering it as a protocol.

The development of laser technology and the potential antimicrobial effects when combined with a dye solution (known as photodynamic therapy) have introduced this treatment modality as a possible coadjuvant in the treatment of periodontitis [29]. As observed in the present clinical case, the association of low power lasers and an appropriate restorative treatment can bring benefits for biological tissues, improve prosthetic procedures, and provide the patient with a more comfortable and predictable treatment.

## 4. Conclusion

The therapy with low power laser can contribute positively to the final outcome of indirect restorative treatment, reducing gingival inflammatory processes. Nevertheless, it is important that the patient should practice a strict oral hygiene routine. Due to the different interactions of lasers with biological tissues, the knowledge of their correct use is of extreme importance for clinical success.
